# Growth and Nutrient Utilization in Basil Plant as Affected by Applied Nutrient Quantity in Nutrient Solution and Light Spectrum

**DOI:** 10.3390/biology11070991

**Published:** 2022-06-29

**Authors:** Xiaowei Ren, Na Lu, Wenshuo Xu, Yunfei Zhuang, Satoru Tsukagoshi, Michiko Takagaki

**Affiliations:** 1 Graduate School of Horticulture, Chiba University, 648 Matsudo, Matsudo City 271-8510, Chiba, Japan; xwrnjau@gmail.com (X.R.); xuwenshuo1988@163.com (W.X.); zhuangyunfei1015@outlook.com (Y.Z.); mtgaki@faculty.chiba-u.jp (M.T.); 2 Center for Environment, Health, and Field Sciences, Chiba University, 6-2-1 Kashiwanoha, Kashiwa 277-0882, Chiba, Japan; tsukag@faculty.chiba-u.jp

**Keywords:** hydroponics, quantitative nutrient management, plant factory, LEDs, red:blue ratio, nutrient use efficiency

## Abstract

**Simple Summary:**

The growth and nutrient utilization of hydroponic vegetables are largely affected by the nutrient solution management method and light environment. This study was conducted to improve the growth, nutrient absorption efficiency, and nutrient use efficiency of basil plants grown in a plant factory with artificial lighting by controlling the nutrient solution management method and light spectrum. Basil plants were treated with 4 applied nutrient quantities and three red:blue ratios from transplanting to harvest (20 days). Results showed that low applied nutrient quantity significantly improved the nutrient use efficiency and nutrient absorption efficiency. Furthermore, the yield of the basil plant and the absorption of N and K were significantly influenced by different red:blue ratios under low applied nutrient quantity treatments. Therefore, this study has determined the optimal combination of the applied nutrient quantity and red:blue ratio for improving the growth, nutrient use efficiency, and nutrient absorption efficiency of basil plants. The findings of this study can be applied to hydroponic basil production for saving resources and protecting the environment.

**Abstract:**

Quantitative nutrient management has advantages, such as saving resources and improving nutrient utilization, compared with the conventional electrical conductivity management method. The growth and nutrient utilization of vegetables are affected by the integrated environmental conditions such as nutrient supply and light spectrum. This study investigated the effects of applied nutrient quantity (ANQ) (0.5, 1, 2, and 4 times (T) the absorption quantity of nutrients determined in the preliminary experiment, indicated by 0.5T, 1T, 2T, and 4T, respectively) in nutrient solution and red:blue ratio (R:B = 3:7, 7:3, and 9:1, indicated by RB3:7, RB7:3, and RB9:1, respectively) on the growth and nutrient utilization of basil plants in a plant factory with artificial lighting. Results demonstrated that the nutrient use efficiency (NUE) and the nutrient absorption efficiency (NAE) were significantly increased by the ANQ of 0.5T compared with the treatments of 1T, 2T, and 4T, irrespective of R:B ratios. Furthermore, under the ANQ of 0.5T, RB7:3 significantly increased the yield and the absorption of N and K of the basil plant compared with other R:B ratios. Therefore, the ANQ of 0.5T combined with RB7:3 was considered the optimal combination to improve the yield, NUE, and NAE of basil plants in the present study.

## 1. Introduction

The world is increasingly facing the problem of resource shortage [1], and hence, it is particularly important to improve the efficiency of resource use. In agricultural production, the excessive use of nutrients is a factor limiting the sustainable development of agriculture [2]. Hydroponics is an important vegetable cultivation method in protected agriculture, and the nutrient solution (NS) used in hydroponics can be recycled, which significantly improves the water and nutrient use efficiency (NUE). However, the accumulation of vegetable root exudates and nutrient imbalance leads to the reduction in vegetable yield under long-term recycled NS (18 days for lettuce and 4 months for tomato) [3,4]. Additionally, it is difficult to remove the root exudates and balance the nutrients. Therefore, growers prefer to discard the recycled NS [3]. This discard of NS into the natural environment will cause environmental pollution and resource waste [5]. Specifically, the discard of a large amount of PO_4_^3−^ into a water body results in eutrophication [6,7]. Moreover, phosphate nutrient waste will exacerbate phosphate rock depletion [8]. Nitrogen in NS primarily exists in the form of NO^3−^ and NH_4_^+^, and NH_4_^+^ is converted into nitrate ions by the nitrification of microorganisms in soil [9]. These NO^3^^−^ ions will enter the groundwater and cause excessive nitrate concentration, which is harmful to the human body [9]. Furthermore, catalytic ammonia synthesis consumes an excessive amount of energy [10], which indicates that ammonium waste leads to excessive energy waste. K^+^ is an important source of groundwater salinization in the semi-arid context [11]. Na^+^ may cause the dispersion of soil particles and the deficiency or imbalance of soil nutrients, thereby affecting plant growth [12]. Therefore, protecting the environment and saving resources by improving the absorption and use efficiency of nutrients are highly significant aspects.

The electrical conductivity (EC) of NS can be easily measured, and by monitoring the EC of NS, the total amount of available ions in NS can be estimated [13]. Furthermore, the growth and quality of vegetables are significantly influenced by the EC of NS [14,15,16]. Consequently, EC management is a commonly used NS management method in hydroponic vegetable production [17]. Nonetheless, there are also some disadvantages of the EC management method. For instance, the EC value does not reflect the specific ion content in the NS after a period of use for plant cultivation [3]. Moreover, long-term control of EC at a target value may lead to nutrient imbalance due to the selective absorption of nutrients by plants. On the one hand, vegetables often absorb certain nutrients (e.g., NO^3^^−^, PO^4^^−^, and K^+^) more than their requirement when fertilizers are supplied continuously to maintain the EC of NS constant. The plant yield will no longer increase when the absorption of these nutrients exceeds a threshold (luxury absorption), which will lead to resource waste [18,19]. On the other hand, Ca^2^^+^ and Mg^2^^+^ will be accumulated in the NS due to the relatively weaker absorption capacity of plants [20]. Therefore, even if the EC value of NS reaches the target, the balance of ions becomes no longer suitable for plant growth [3].

To compensate for the limitations of the EC management method, a new NS management method, quantitative nutrient management (QNM), was developed [21]. In QNM, nutrients are supplied to the NS quantitatively and regularly, irrespective of the EC value of NS [19,22]. Although QNM fluctuates the concentration of ions, the yield of lettuce was not significantly affected, and most ions were absorbed [5]. Furthermore, QNM enabled avoiding the luxury absorption of nutrients and increased nutrient utilization because the nutrients could be supplemented according to the nutrient requirements of spinach [23]. QNM is more beneficial than the EC management method for the future development of hydroponics in terms of saving resources and regulating vegetable quality. In recent years, QNM has been applied to control the yield and quality of vegetables [5,24].

The applied nutrient quantity (ANQ) has a significant impact on the growth and nutrient absorption (NA) of vegetables. The shoot dry weight of tomato was decreased with the decrease in ANQ [25]. In another study, the leaf dry weight of tomato remained unaffected by different applied potassium quantities, and the potassium absorption increased with the increase in applied potassium quantity [24]. The shoot fresh and dry weights of lettuce were decreased with the decrease in applied nitrate quantity, and the nitrate absorption decreased with the decrease in applied nitrate quantity [5].

A plant factory with artificial lighting (PFAL) is a closed system used to produce valuable plants such as vegetables and herbs [26]. Additionally, PFALs improve the nutrient, water, and land-use efficiency compared with traditional agriculture [27]. The growth and NA of vegetables can be controlled not only by nutrient supply but also by light spectrum in a PFAL. Red and blue light are the main energy sources for plant photosynthesis, and the combination of red light and blue light is widely used as a light source for vegetable production in PFALs [26,28,29]. A review article has summarized the effects of the light spectrum on nutrient acquisition and utilization of different crops and highlighted that red light promoted the absorption of N in Chinese chive, Ca in gynostemma, P and K in cucumber, and Zn in spinach, while blue light promoted the absorption of P and K in Chinese chive, N, P, and K in garlic, and N and Ca in spinach [30]. Different red:blue (R:B) ratios also exert significant effects on the growth and NA of vegetables. The shoot fresh weight of the basil plant was found to be higher at R:B ratios of 2:1, 3:1, and 4:1 than at R:B ratios of 1:2 and 1:1 [26]. The shoot fresh and dry weights of lettuce were also found to be higher at an R:B ratio of 3:1 than those at R:B ratios of 1:2, 1:1, and 2:1 [31]. Moreover, an R:B ratio of 9:2 produced higher shoot fresh and dry weights of lettuce than other R:B ratios [32]. In the shoot of broccoli, an R:B ratio of 4:1 increased the concentration of Ca, Mg, P, and S; however, the shoot fresh and dry weights were not influenced by different R:B ratios [33]. Other studies have reported that the accumulation of N, P, K, and Mg in basil [26] and in lettuce [31] were promoted by an R:B ratio of 3:1 (among the R:B ratios of 1:2, 1:1, 2:1, 3:1, and 4:1).

These studies focused on the effects of nutrient supply and red light, blue light, or R:B ratios on the yield, nutrition concentration, and nutrition accumulation of different plants, but there are still no data on the effects of the combination of ANQ and R:B ratios on the total NA, NA efficiency (NAE), and NUE. From their results, we hypothesized that an R:B ratio between 2:1 and 4:1 could promote the yield of basil, which is one of the major herbs grown in PFALs. Different R:B ratios could also significantly affect the NA of macro-elements in NS, especially under different ANQs. In the present study, a low ANQ with an R:B ratio that maximizes yield is aimed to be determined to improve the NAE and NUE of basil plants in a PFAL. The results could expand our understanding of the association between nutrient uptake and light spectrum in basil plants and help the producers to produce basil more effectively and economically. 

## 2. Materials and Methods

### 2.1. Plant Material and Plant Cultivation

Sweet basil (*Ocimum basilicum* L. var. *basilicum* L. cv. Genovese, Takii & Co., Ltd., Kyoto, Japan) seeds were sown in sponge cubes (2.3 × 2.3 × 2.8 cm, 14.8 cm^3^) placed in germination boxes (22 × 14 × 5 cm, 1.54 L) at a temperature of 20 °C for 48 h for germination. The sponge cubes were soaked in an NS (N 22.20 mM, P 2.04 mM, K 9.12 mM, Mg 2.22 mM, Ca 4.92 mM, Fe 61.07 μM, Cu 0.38 μM, Zn 0.73 μM, Mo 0.25 μM, Mn 16.80 μM, and B 35.56 μM, under an EC of 3.0 dS m^−1^) (Otsuka hydroponic composition, OAT Agrio Co., Ltd., Tokyo, Japan). The EC and pH of the NS were adjusted to 1.8 dS m^−1^ and 6.5, respectively. After germination, the seedlings were placed under white light-emitting diode (LED) lamps with a photosynthetic photon flux density (PPFD) of 200 ± 15 μmol m^−2^ s^−1^. The spectrum of the white LED lamp is shown in Supplementary Figure S1. At 20 days after sowing, two experiments were conducted. Experiment 1 was to determine the daily NA of the basil plant under the optimal EC condition (EC = 3 dS m^−1^) confirmed by our previous study [34] for designing the ANQ of the control in Experiment 2. Experiment 2 was to investigate the effects of different ANQ and R:B ratios on the growth and nutrient utilization of basil plants.

**Figure 1:**
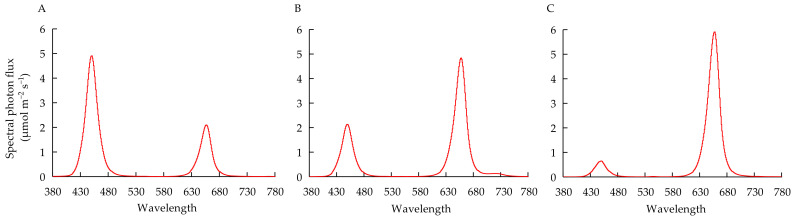
Spectral distribution of the LED lamps of RB3:7 (**A**) RB7:3 (**B**) and RB9:1 (**C**) used in the experiment.

In Experiment 1, the seedlings were transplanted to a cultivation box (32 × 18 × 9 cm, 5.18 L) containing 3 L NS (EC = 3 dS m^−1^) till harvest. The NS volume and EC value of NS were adjusted to 3 L and 3 dS m^−1^, respectively, every 2 days. Simultaneously, the pH was adjusted to 6.5. The plant density was 100 plants m^−2^ [35]. The nutrient concentration was the same as described earlier. Light was provided using the LED lamps (GreenPower research modules, Philips, Pila, Poland) with a PPFD of 200 ± 15 μmol m^−2^ s^−1^ (R:B ratio of 7:3). The light spectrum is shown in Figure 1B. The temperature, photoperiod, CO_2_ concentration, and relative humidity were set to 24 °C/21 °C (day/night), 16/8 h (day/night), 500 ppm, and 65–80%, respectively. The NS was sampled on the 10th, 15th, and 20th days after transplanting to determine the absorption of nutrients by the plants during 1–10, 11–15, and 16–20 days after transplanting. 

In Experiment 2, the seedlings were subjected to 4 × 3 = 12 treatments in total. Two factors, namely, quantitative fertilization mode and light spectrum, were included in this experiment. For the quantitative fertilization mode, 4 levels of nutrient element quantity, 0.5, 1, 2, and 4 times the absorption quantity of nutrients determined in Experiment 1 (hereafter indicated by 0.5T, 1T, 2T, and 4T, respectively), were supplied during transplanting to harvest. The addition of nutrients was divided into three stages. The first stage was 1–10 days after transplanting, the second stage was 11–15 days after transplanting, and the third stage was 16–20 days after transplanting. The NS volume was adjusted to 3 L every 2 days, and simultaneously, the pH was adjusted to 6.5. The concentrated NS was added quantitatively on the 1st, 11th, and 16th days after transplanting. The fertilization design is shown in Table 1. For light spectra, 3 different R:B ratios, 3:7, 7:3, and 9:1 (hereafter indicated by RB3:7, RB7:3, and RB9:1, respectively), were used. The proportion of red light was calculated by defining the relative areas of the spectrum within the red light region [36]. The light was provided by dimmable red and blue LED lamps (GreenPower research modules, Philips, NL). Six red LED lamps and six blue LED lamps were used in each treatment. The target R:B ratio was obtained by dimming the light intensity of red and blue LED lamps and was measured using a spectrum meter (Lighting Passport Pro, ALP-01, ASENSETEK INC., Taipei, Taiwan). Moreover, the total PPFD at the surface of planting panels was adjusted to 200 ± 15 μmol m^−2^ s^−1^. RB3:7, RB7:3, and RB9:1 indicate that the light intensities of red light and blue light were 60 and 140 μmol m^−2^ s^−1^, 140 and 60 μmol m^−2^ s^−1^, and 180 and 20 μmol m^−2^ s^−1^, respectively. The treatment of 1T under RB7:3 was used as the control. The light spectra of different R:B ratios are shown in Figure 1. The temperature, photoperiod, CO_2_ concentration, and relative humidity were set to 24 °C/21 °C (day/night), 16/8 h (day/night), 500 ppm, and 65–80%, respectively. From transplanting to harvest, the NS was sampled on the 10th, 15th, and 20th days to detect the contents of each nutrient element.

### 2.2. Measurements

#### 2.2.1. Plant Growth Parameters

The shoot, leaf, stem, and root fresh weights of sweet basil plants were measured on harvest day (20 days after transplanting). Then, they were oven-dried at 80 °C for 3 days to measure the dry weights. Six replicates were performed for each treatment.

The total leaf area was measured using a leaf area meter (LI–3000, Li-Cor, Lincoln, NE, USA). The leaf dry mass per area (LMA) was calculated as the leaf dry weight divided by leaf area. Six replicates were performed for each treatment.

#### 2.2.2. Gas Exchange Parameters

At 20 days after transplanting, the gas exchange parameters were determined using a gas exchange system (LI-6400, Li-Cor, Inc., Lincoln, NE, USA) and the integrated fluorescence chamber head (LI-6400-40, Li-Cor, Inc., Lincoln, NE, USA). Three replicates were performed for each treatment. The third pair of leaves from the top was used for the measurement of each parameter. The PPFD, CO_2_ concentration, relative humidity, and air temperature inside the leaf chamber were set at 200 µmol m^−2^ s^−1^, 500 mmol mol^−1^, 65%, and 22 °C, respectively. The R:B ratios during measurement were set to 3:7, 7:3, and 9:1, respectively.

#### 2.2.3. Chlorophyll Fluorescence Parameters

The chlorophyll fluorescence parameters of basil plants were determined on harvest day according to previously described methods [37]. The third pair of leaves from the top was used for the measurement of each parameter.

#### 2.2.4. NA, NUE, NAE, and Nutrient Waste (NW) from Producing 1 g of Shoot Dry Weight

The nutrient content was measured using an ion chromatography system (ICS-1100, Thermo Fisher Scientific, Inc., Tokyo, Japan).

The NA, NUE [38], NAE [21], and NW were calculated as follows:

NA = TAN − FAN

where TAN is the total amount of applied nutrients and FAN is the final amount of nutrients remaining.

NUE = SDW/NA

where SDW is the shoot dry weight.

NAE = NA/TAN

NW = FAN/SDW

### 2.3. Statistical Analysis

The mean values of data were compared using Tukey’s test in the SPSS statistical software (IBM SPSS Statistics, Version 19.0. Armonk, NY, USA: IBM Corp.). A *p*-value of < 0.05 was used to determine whether the difference was significant.

## 3. Results

### 3.1. Experiment 1

#### Daily Absorption of Nutrients by Basil Plants

The daily absorption of NO^3−^, PO_4_^3−^, K^+^, Ca^2+^, Mg^2+^, and SO_4_^2−^ by basil plants increased with the stage of the plant. Basil plants showed the largest daily absorption of NO^3−^, followed by K^+^, PO_4_^3−^, Ca^2+^, SO_4_^2−^, and Mg^2+^ (Table 2).

### 3.2. Experiment 2

#### 3.2.1. Comparison of Plant Growth between Experiment 1 and the Control Group (1T under RB7:3) in Experiment 2

The shoot and leaf fresh and dry weights of basil plants showed no significant differences between Experiment 1 and the control group in Experiment 2 (Table 3).

#### 3.2.2. Plant Growth under Four ANQ Treatments and Three R:B Ratios

**Figure 2:**
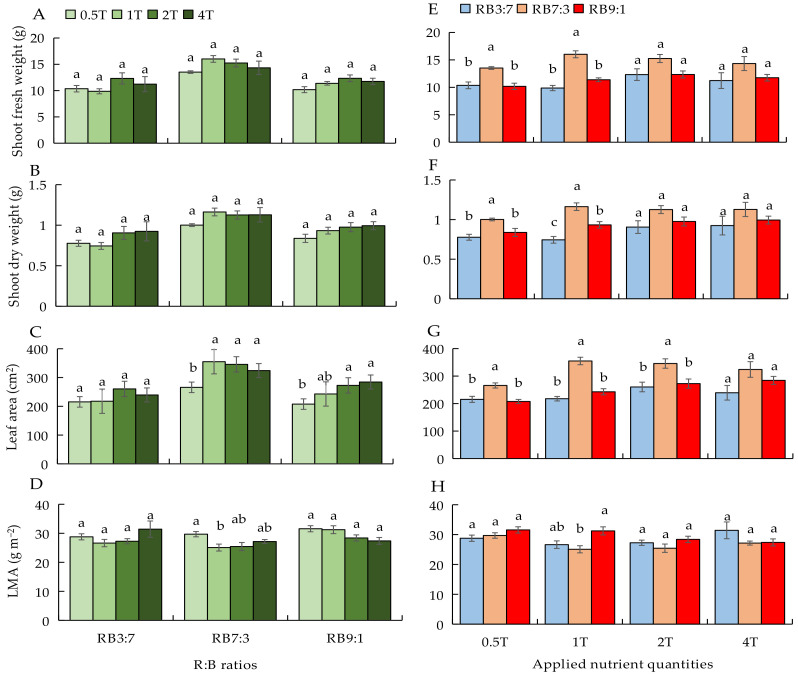
Shoot fresh (**A**,**E**) and dry weights (**B**,**F**), leaf area (**C**,**G**), and leaf dry mass per area (LMA) (**D**,**H**) at 20 days after transplanting under different ANQ treatments and R:B ratios. 0.5T, 1T, 2T, and 4T represent 4 different ANQ treatments, respectively. RB3:7, RB7:3, and RB9:1 represent R:B ratios of 3:7, 7:3, and 9:1, respectively. The error bars represent SEs (n = 6). Based on Tukey’s new multiple range test at *p* < 0.05. Different lowercase letters represent significant differences among different treatments.

To describe the results more clearly, we used the same data to plot two types of graphs in different forms (left and right). As observed on the left side of Figure 2, there was no difference in all growth parameters at different ANQ treatments under RB3:7 (Figure 2A–D). Under RB7:3, the shoot fresh and dry weights were not affected by different ANQ treatments; however, the leaf area at 0.5T was significantly lower than at other ANQ treatments. The LMA at 0.5T was significantly higher than that at 1T, but there was no significant difference in LMA among 1T, 2T, and 4T (Figure 2A–D). Under RB9:1, the shoot fresh and dry weights were not significantly different among different ANQ treatments. The leaf area was lower at 0.5T than at 2T and 4T. The difference in LMA under different ANQ treatments was not significant (Figure 2A–D).

As observed in the right side of Figure 2, under 0.5T, the shoot fresh and dry weights and leaf area were significantly higher at RB7:3 than at RB3:7 and RB9:1 (Figure 2E–G); however, the differences in LMA at different R:B ratios were not significant (Figure 2H). The shoot fresh and dry weights and leaf area at different R:B ratios under 1T exhibited a similar trend as those parameters at different R:B ratios under 0.5T; however, the LMA at RB9:1 was significantly higher than that at RB7:3 (Figure 2E–H). Under 2T, the leaf area at RB7:3 was significantly higher than that at RB3:7 and RB9:1, but the other growth parameters showed no significant differences among different R:B ratios (Figure 2E–H). No significant difference was observed in all parameters among different R:B ratio treatments under 4T (Figure 2E–H).

The effects of different ANQ treatments and R:B ratios on the leaf fresh and dry weights and root fresh and dry weights are shown in Supplementary Table S1.

The different ANQ treatments exerted no significant effects on leaf fresh and dry weights under RB3:7 and RB7:3, but the leaf fresh weight was higher at 2T than at 0.5T under RB9:1. The leaf fresh and dry weights were higher at RB7:3 than at RB3:7 and RB9:1 under 0.5T and 1T, but the differences in leaf fresh and dry weights among different R:B ratios were not significant under 2T and 4T (Table S1). The different ANQ treatments had no significant impact on the root fresh weight under RB3:7 and RB9:1; however, the root fresh weight was higher at 1T than at 0.5T under RB7:3. The different R:B ratios had no significant impact on the root fresh weight under 0.5T, 2T, and 4T, but the root fresh weight was higher at RB7:3 than at RB9:1 under 1T. The root dry weight was not affected by different ANQ treatments, irrespective of R:B ratios. The root dry weight was higher at RB7:3 than at other R:B ratios under 0.5T, but there was no significant difference in the root dry weight among different R:B ratios under other ANQ treatments (Table S1).

#### 3.2.3. Gas Exchange and Chlorophyll Fluorescence Parameters

Net photosynthetic rate, transpiration rate, electron transport rate, maximum quantum yield of PSII primary photochemistry, efficiency of excitation energy captured by open PSII reaction centers, quantum yield of PSII electron transport, photochemical quenching, and nonphotochemical quenching were not affected by the ANQ treatments and R:B ratios (Table 4).

The correlation between shoot dry weight, leaf area, and photosynthesis parameters is shown in Supplementary Table S2. There was a strong positive correlation between shoot dry weight and leaf area. However, there was no correlation between the shoot dry weight and photosynthesis parameters (Table S2).

#### 3.2.4. Absorption and Utilization of N, P, and K under Different ANQ Treatments

**Figure 3:**
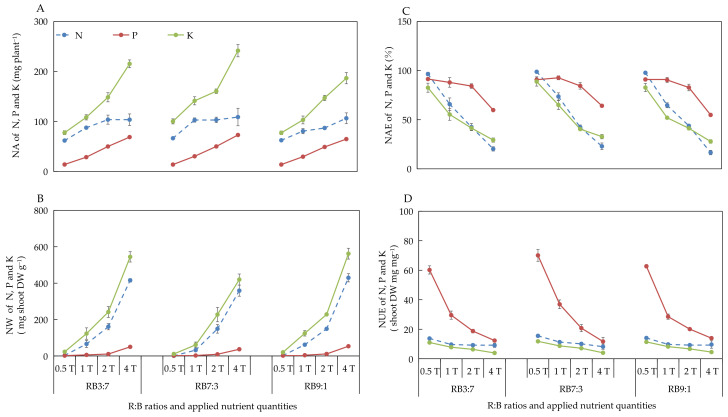
Nutrient absorption (NA) (**A**), nutrient waste (NW) (**B**) from producing 1 g shoot dry weight, nutrient absorb efficiency (NAE) (**C**), and nutrient use efficiency (NUE) (**D**) of shoot dry weight of N, P, and K after 20 days of cultivation under different ANQ treatments and R:B ratios. 0.5T, 1T, 2T, and 4T represent 4 different ANQ treatments. RB3:7, RB7:3, and RB9:1 represent the R:B ratios of 7:3, 3:7, and 9:1, respectively. The error bars represent SEs (n = 3).

The different ANQ treatments exerted a significant effect on the absorption and utilization of N, P, and K, irrespective of the R:B ratios. Furthermore, the absorption and utilization of N, P, and K by the basil plant showed a clear trend under different ANQ treatments, irrespective of the R:B ratios. Specifically, the NA and NW of N, P, and K increased with the increase in ANQ (Figure 3A,B). The NAE and NUE of N, P, and K decreased with the increase in ANQ (Figure 3C,D).

#### 3.2.5. Absorption and Utilization of Ca, Mg, and S under Different ANQ Treatments

**Figure 4:**
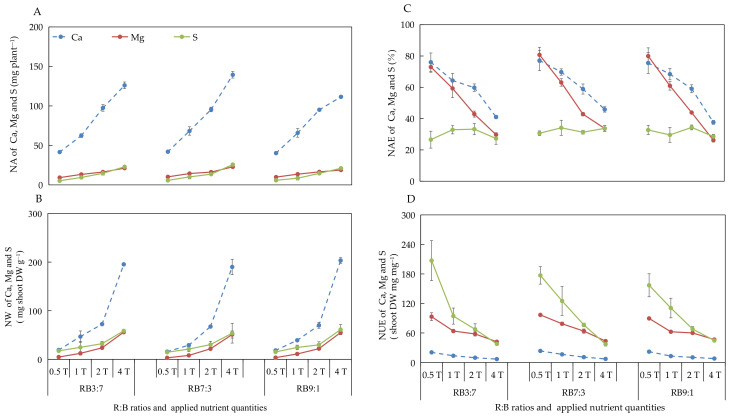
Nutrient absorption (NA) (**A**), nutrient waste (NW) (**B**) from producing 1 g shoot dry weight, nutrient absorb efficiency (NAE) (**C**), and nutrient use efficiency (NUE) (**D**) of shoot dry weight of Ca, Mg, and S after 20 days of cultivation under different ANQ treatments and R:B ratios. 0.5T, 1T, 2T, and 4T represent 4 different ANQ treatments. RB3:7, RB7:3, and RB9:1 represent the R:B ratios of 7:3, 3:7, and 9:1, respectively. The error bars represent SEs (n = 3).

The effect of different ANQ treatments on the absorption and utilization of Ca, Mg, and S were consistent with that of N, P, and K, with the only difference being the effect on the NAE of S. The different ANQ treatments exerted no significant effect on the NAE of S, irrespective of the R:B ratios (Figure 4).

#### 3.2.6. Absorption and Utilization of N, P, and K at Different R:B Ratios

**Figure 5:**
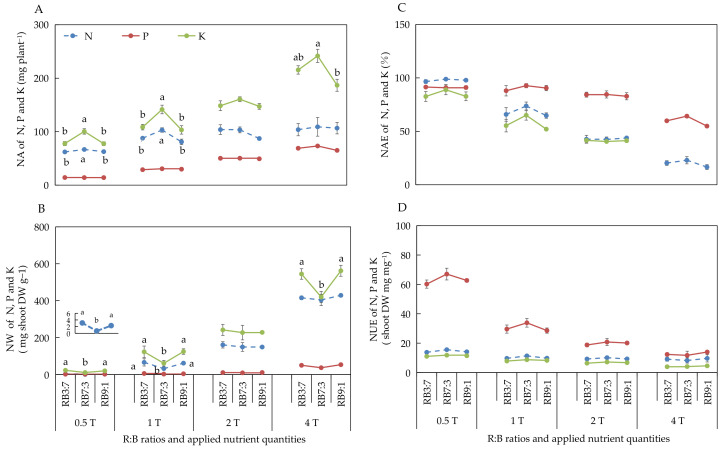
Nutrient absorption (NA) (**A**), nutrient waste (NW) (**B**) from producing 1 g shoot dry weight, nutrient absorb efficiency (NAE) (**C**), and nutrient use efficiency (NUE) (**D**) of shoot dry weight of N, P, and K after 20 days of cultivation under different ANQ treatments and R:B ratios. 0.5T, 1T, 2T, and 4T represent 4 different ANQ treatments. RB3:7, RB7:3, and RB9:1 represent the R:B ratios of 7:3, 3:7, and 9:1, respectively. The small graph in Figure 5B represents the NW of N at different R:B ratios under 0.5T. The error bars represent SEs (n = 3). On the basis of Tukey’s new multiple range test at *p* < 0.05. Different lowercase letters represent significant differences among different treatments.

The NA of N and K was significantly increased at RB7:3 compared with that at other R:B ratios under 0.5T and 1T (Figure 5A). However, the NA of N and K remained unaffected at different R:B ratios under 2T. The NA of N remained unaffected at different R:B ratios under 4T. However, the NA of K was significantly higher at RB7:3 than at RB9:1, but there was no significant difference in the NA of K between RB3:7 and RB7:3 under 4T. There was also no significant difference in the NA of P among different R:B ratios, irrespective of the ANQ treatments (Figure 5A).

The NW of N and K was significantly decreased at RB7:3 compared with that at other R:B ratios under 0.5T and 1T. However, these parameters were not affected at different R:B ratios under 2T. Furthermore, the NW of N remained unaffected at different R:B ratios under 4T. The NW of K was significantly lower at RB7:3 than at other R:B ratios under 4T. No significant difference was observed in the NW of P among different R:B ratios, irrespective of the ANQ treatments (Figure 5B). The NAE and NUE of N, P, and K were not affected at different R:B ratios, irrespective of the ANQ treatments (Figure 5C,D).

#### 3.2.7. Absorption and Utilization of Ca, Mg, and S at Different R:B Ratios

**Figure 6:**
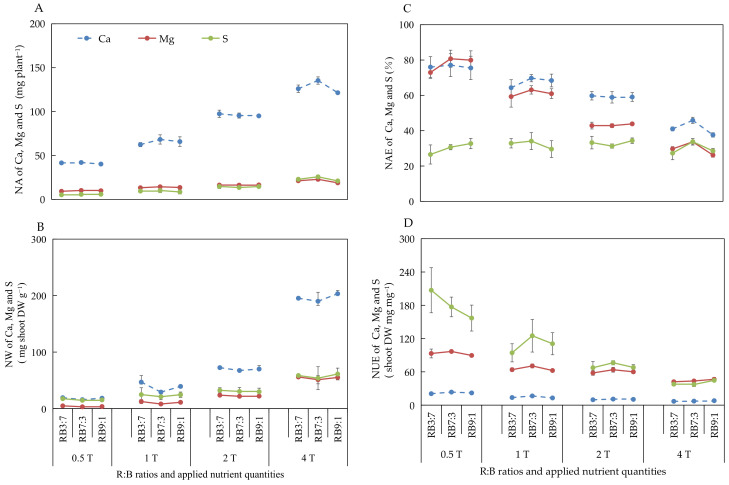
Nutrient absorption (NA) (**A**), nutrient waste (NW) (**B**) from producing 1 g shoot dry weight, nutrient absorb efficiency (NAE) (**C**), and nutrient use efficiency (NUE) (**D**) of shoot dry weight of Ca, Mg, and S after 20 days of cultivation under different ANQ treatments and R:B ratios. 0.5T, 1T, 2T, and 4T represent 4 different ANQ treatments. RB3:7, RB7:3, and RB9:1 represent the R:B ratios of 7:3, 3:7, and 9:1, respectively. The error bars represent SEs (n = 3).

The NA of Ca tended to increase with the increase of the R:B ratios from 3:7 to 7:3 and then decrease when the R:B ratios increased from 7:3 to 9:1 under 1T and 4T. The NW of Ca showed the opposite tendency of NA under the same treatments. There were no obvious changes in the NA and NW of Ca at different R:B ratios under 0.5T and 2T. Moreover, there were no obvious changes observed in the NA and NW of Mg and S at different R:B ratios, irrespective of the ANQ treatments (Figure 6A,B). 

The NAE of Mg tended to increase with the increase of the R:B ratios from 3:7 to 7:3 and then decrease when the R:B ratios increased from 7:3 to 9:1 under 0.5T, 1T, and 4T. The changes of NAE in Ca showed similar trends as Mg under 1T and 4T. The NAE of S showed different patterns with the change in the R:B ratios under different ANQ treatments, whereas there were no significant differences observed. There were no changes in the NAE of Ca, Mg, and S at different R:B ratios under 2T (Figure 6C). 

The NUE of S showed a decreasing trend with the increase of R:B ratios under 0.5T, whereas it tended to first increase and then decrease with the increase of R:B ratios under 1T. Moreover, the NUE of Ca and Mg were not affected at different R:B ratios, irrespective of the ANQ treatments (Figure 6D).

## 4. Discussion

### 4.1. Growth Response of Basil Plant to ANQ Treatments and R:B Ratios

In the present study, 1T was the amount of nutrients absorbed by the basil plant at the optimal EC (EC = 3.0 dS m^−1^) for basil growth, as confirmed by our previous study [34]. Furthermore, there were no significant differences in shoot fresh and dry weights and leaf fresh and dry weights between the EC management method and QNM (Table 3), which indicated that 1T had fulfilled the nutrient requirements of basil growth. When the amount of nutrients absorbed by plants exceeds a threshold (luxury absorption), the plant yield no longer increases with the increase in NA. For instance, with the increase in the EC of NS, the absorption of some macronutrients (N, P, K, and Ca) by lettuce was found to be increased, but there was no increase in the yield of lettuce [18]. In our study, although the NA increased with the increase in ANQ from 1T to 4T, the shoot fresh and dry weights did not increase with the increase in ANQ (Figure 2A,B), which indicated that 2T and 4T may cause the luxury absorption of nutrients by basil plants. Moreover, an insufficient amount of nutrients can result in a nutrient deficiency that limits plant growth and development [39]. In the present study, 0.5T did not significantly decrease the yield of basil plants compared with 1T (Figure 2A,B), which confirmed that 0.5T did not cause nutrient deficiency for basil growth.

In the present study, the shoot fresh and dry weights of basil plants were significantly higher at RB7:3 than at RB3:7 and RB9:1 under 0.5T and 1T. Furthermore, there were no significant differences in shoot fresh and dry weights among different R:B ratios under 2T and 4T (Figure 1E,F), which indicates that the response of basil growth to the same R:B ratios was regulated by different ANQ treatments. Different R:B ratios caused differences in basil biomass under 0.5T and 1T in our study. However, the net photosynthetic rate and chlorophyll fluorescence parameters were not affected by the different R:B ratios significantly (Table 4). The photosynthetic rate in basil and strawberry (blue light percentage from 7.3 to 37.7%) [36] and the chlorophyll fluorescence parameters (Fv/Fm, PhiPSII, and Fv/Fm-PhiPSII) in cucumber (blue light percentage from 0 to 100%) [40] were significantly affected by R:B ratios when the proportion of blue light was below 10%. In the present study, the proportion of blue light is equal to or greater than 10%, which may be a possible factor leading to these results. Moreover, the leaf area was significantly higher at RB7:3 than at RB3:7 and RB9:1 under 0.5T and 1T (Figure 2G), and a significant positive correlation between shoot dry weight and leaf area was also found (Table S2). Hence, different R:B ratios influenced the leaf area of the basil plant, and thus, the photoassimilates of the entire plant were influenced. Another study also reported that different light spectra affected the interception of light by affecting the leaf area of green basil, resulting in different yields [41]. Besides, it is reported that the fluorescence Fv/Fm of 3 basil cultivars was not affected by different electrical conductivities (ECs) of 1.0, 2.0, and 3.0 dS m^−1^ [42], and the photosynthetic rate of basil was not influenced by different ECs of 0.5, 1.0, 3.0, and 5.0 dS m^−1^ [34], indicating that the photosynthesis of basil is not easily affected by the nutrient amount in the nutrient solution in the ranges used. Moreover, N and P are considered very important for photosynthesis because photosynthesis is a highly regulated process that is coordinately operative with N metabolism [43] on the one hand. On the other hand, carbon is fixed through the photosynthetic carbon reduction cycle in the chloroplast, and the fixed carbon combines with the phosphate as triose phosphate (triose-P) in the cytosol, then the triose-P is converted to sucrose [44]. Therefore, photosynthesis would be affected if N and P supplies were not enough for plants. The photosynthesis parameters were not affected by different ANQs, which indicated that there was no N and P deficiency for basil plants in the ranges used in the present study.

### 4.2. Nutrient Utilization under ANQ Treatments and R:B Ratios

Different ions have different transport mechanisms. For example, the uptake of NO^3^^−^, PO^4^^−^, and SO_4_^2^^−^ is driven by cotransport with H^+^. The uptake of Ca^2+^ and K^+^ occurs via channels (transmembrane proteins) [45]. Although the transport mechanisms are different for different ions, the electrochemical potential gradient is the driving force for ion transport [45,46]. Differences in ion concentration inside and outside root cells create different electrochemical potential gradients [47]. High ANQ could result in high ion concentration and high electrochemical potential gradients, and, thus, high ANQ promoted nutrient absorption in the present study (Figure 3A and Figure 4A). In general, the absorption of ions by plants is mainly affected by two factors, ion concentration and the number of transport proteins in the roots [48]. It usually increases with increasing ion concentration firstly and stops increasing when the ion concentration reaches a certain high level due to the limitation of the number of transport proteins on the root cell membrane. In the present study, the absorption of ions by basil plants kept increasing with the increase in ANQ under all light conditions, indicating that the ion concentration was the main factor responsible for the increase in ion absorption. 

In our study, the absorption of N, P, K, Ca, Mg, and S by the basil plant increased with the increase in ANQ at all three R:B ratios (Figure 3A and Figure 4A). However, the shoot fresh and dry weights showed no increase with the increase in ANQ. Similarly, in another study, the absorption of K by tomato was increased with the increase in applied potassium quantity; however, the leaf dry weight of tomato did not increase with the increased absorption of potassium [24]. The absorption of excess nutrients that cannot be converted into biomass is known as luxury absorption [18,19], which causes the waste of nutrients. In this study, 0.5T minimized the NA without sacrificing the yield of the basil plant, which significantly reduced the wastage of nutrients due to luxury absorption. Moreover, although the NA of the basil plant increased with the increase in ANQ, the ratio of NA to ANQ decreased with the increase in ANQ, which led to a decrease in the NAE of the basil plant for N, P, K, Ca, and Mg with the increase in ANQ (Figure 3C and Figure 4C).

Regarding NW, the shoot dry weight of the basil plant remained unaffected by the ANQ treatments. However, the FAN in the NS after harvest increased with the increase in ANQ, which resulted in high NWs under high ANQ treatments. Therefore, an ANQ of 0.5T minimized the NW, especially for N, K, Ca, Mg, and S, compared with other ANQ treatments (Figure 3B and Figure 4B). For instance, the NW of N and K at 1T was more than 5 times that at 0.5T. The NW of Ca, Mg, and S at 1T was more than 2 times that at 0.5T. Therefore, 0.5T significantly reduced the waste of nutrients and environmental pollution.

NUE represents the ability of a plant to convert absorbed nutrients into biomass [49], and there are 3 methods to improve NUE, as follows: (1) The yield remains unchanged, and the absorption of nutrients is reduced; (2) The yield increases and NA remains unchanged; (3) The increase in the rate of yield is more than the rate of increase in the absorption of nutrients [50]. In the present study, the shoot dry weight of the basil plant was not significantly affected by the different ANQ treatments, and the absorption of N, P, K, Ca, Mg, and S was reduced with the decrease in ANQ treatments, which resulted in the increase in the NUE of N, P, K, Ca, Mg, and S with the decrease in ANQ treatments (Figure 3D and Figure 4D).

There were significant differences in the NA and NW of N and K among different R:B ratios in the present study. The transpiration rate was not affected by different R:B ratios (Table 4). However, the leaf area of the basil plant was significantly higher at RB7:3 than at other R:B ratios under 0.5T and 1T, which led to higher transpirational fluxes of the entire basil plant at RB7:3 than at other R:B ratios. The increased transpirational fluxes may be the reason for the high N and K absorption in the present study because NA increased with the increase in transpirational fluxes in wheat [51]. Regarding the NW of N and K, the NA was higher at RB7:3 than at other R:B ratios under 0.5T and 1T, which led to the FAN in the solution tank after the harvest was lower at RB7:3 than at other R:B ratios. Meanwhile, the shoot dry weight was significantly higher at RB7:3 than at other R:B ratios under 0.5T and 1T. The decreased FAN and the increased shoot dry weight led to a significant decrease in the NW of N and K at RB7:3 compared with that at other R:B ratios under 0.5T and 1T.

## 5. Conclusions

This study explored the effects of quantitative nutrient management with different applied nutrient quantities and red:blue ratios on the growth and nutrient utilization of basil plants. Results showed that low applied nutrient quantity significantly increased the nutrient use efficiency and nutrient absorption efficiency and decreased the nutrient waste without decreasing the shoot fresh and dry weights of the basil plant. Furthermore, we observed that RB7:3 was more conducive to basil production than other red:blue ratios under a low applied nutrient quantity condition because RB7:3 promoted plant growth and nutrient absorption of N and K and decreased the nutrient waste of N and K. Therefore, quantitative nutrient management combined with an optimal red:blue ratio could enhance the growth and nutrient utilization of basil plants and reduce resource waste in hydroponic vegetable production in a plant factory with artificial lighting. The quality of the basil plant was not evaluated in the present study. Further studies are required to investigate the effect of the combination of quantitative nutrient management and red:blue ratios on the quality of basil plants in the future.

## Figures and Tables

**Table 1:** Fertilization design of Experiment 2.

Growth Stage (Days)		Quantity of Nutrient Supply (mg Plant^−1^)								
	Applied Nutrient Quantity	Ca(NO_3_)_2_·4H_2_O	KNO_3_	Mg(NO_3_)_2_·6H_2_O	NaNO_3_	K_2_SO_4_	NaH_2_PO_4_	KCl	MgSO_4_·7H_2_O	KH_2_PO_4_
1–10	0.5T	33.50	36.23	19.22	24.00	10.45	14.57	6.17	0.00	0.00
	1T	67.00	72.46	38.44	48.00	20.90	29.13	12.33	0.00	0.00
	2T	134.00	144.91	76.88	96.00	41.81	58.27	24.67	0.00	0.00
	4T	268.00	289.82	153.75	192.00	83.61	116.53	49.33	0.00	0.00
11–15	0.5T	51.24	54.76	3.20	12.75	0.00	0.00	0.00	19.12	15.61
	1T	102.47	109.53	6.41	25.50	0.00	0.00	0.00	38.24	31.23
	2T	204.94	219.05	12.81	51.00	0.00	0.00	0.00	76.48	62.46
	4T	409.89	438.10	25.63	102.00	0.00	0.00	0.00	152.95	124.91
16–20	0.5T	126.12	80.04	16.02	6.38	0.00	0.00	0.00	30.80	32.37
	1T	252.24	160.08	32.03	12.75	0.00	0.00	0.00	61.61	64.74
	2T	504.48	320.15	64.06	25.50	0.00	0.00	0.00	123.21	129.48
	4T	1008.95	640.31	128.13	51.00	0.00	0.00	0.00	246.42	258.96

0.5T, 1T, 2T, and 4T represent the 4 levels of nutrient element amount, 0.5, 1, 2, and 4 times the absorption quantity of nutrients determined in Experiment 1, respectively.

**Table 2:** Daily absorption of NO_3_^−^, PO_4_^3−^, K^+^, Ca^2+^, Mg^2+^, and SO_4_^2−^ by basil plants at 1–10, 11–15, 16–20, and 1–20 days after transplanting in Experiment 1.

Growth Stage	Days	Unit	Nutrients					
			NO_3_^−^	PO_4_^3−^	K^+^	Ca^2+^	Mg^2+^	SO_4_^2−^
Stage 1	1–10	mg plant^−1^ day^−1^	13.33	2.31	4.38	1.12	0.36	1.14
Stage 2	11–15		28.6	4.35	10.32	3.5	0.86	2.86
Stage 3	16–20		50.97	9.01	16.01	8.57	1.78	4.69
Average	1–20		25.56	4.5	8.77	3.58	0.84	2.45

Nutrient solution with an electrical conductivity of 3.0 dS m^−1^ was used for the entire growth period.

**Table 3:** Shoot and leaf fresh weights and shoot and leaf dry weights of basil plants in Experiment 1 and the control group (1T under RB7:3) in Experiment 2.

Growth Parameters	Experiment 1	Control Group in Experiment 2
Shoot fresh weight	16.08 a ^z^	16.02 a
Leaf fresh weight	12.50 a	12.16 a
Shoot dry weight	1.30 a	1.16 a
Leaf dry weight	1.00 a	0.89 a

^z^ Data are shown as means (n = 6). The same letters in each row indicate no significant differences between Experiment 1 and the control group in Experiment 2 at *p* < 0.05, determined by *t*-test.

**Table 4:** Photosynthesis parameters of basil plants grown under different ANQ treatments and R:B ratios.

Photosynthesis	Unit	ANQ	R:B Ratios								
			RB3:7			RB7:3			RB9:1		
Pn	(μmol CO_2_ m^−2^ s^−1^)	0.5T	9.76	±0.70 ^z^	a A	10.32	±1.04	a A	8.99	±0.86	a A
		1T	10.70	±0.30	a A	8.59	±0.90	a A	8.19	±1.68	a A
		2T	11.37	±0.85	a A	9.79	±1.67	a A	8.49	±0.70	a A
		4T	10.00	±0.99	a A	9.66	±1.34	a A	8.73	±0.88	a A
Tr	(mmol m^−2^ s^−1^)	0.5T	1.58	±0.18	a A	1.48	±0.28	a A	1.79	±0.08	a A
		1T	1.47	±0.33	a A	1.48	±0.05	a A	2.08	±0.12	a A
		2T	1.91	±0.18	a A	1.55	±0.07	a A	1.78	±0.18	a A
		4T	1.73	±0.16	a A	1.56	±0.25	a A	1.36	±0.23	a A
ETR	(μmol electrons m^−2^ s^−1^)	0.5T	44.10	±4.04	a A	44.57	±7.33	a A	45.47	±3.21	a A
		1T	44.47	±4.32	a A	37.19	±6.65	a A	39.87	±0.97	a A
		2T	43.19	±3.23	a A	40.59	±3.59	a A	41.61	±5.30	a A
		4T	43.74	±4.69	a A	37.81	±8.26	a A	35.06	±5.81	a A
Fv/Fm		0.5T	0.82	±0.01	a A	0.81	±0.005	a A	0.80	±0.02	a A
		1T	0.82	±0.02	a A	0.81	±0.008	a A	0.80	±0.004	a A
		2T	0.82	±0.01	a A	0.81	±0.009	a A	0.80	±0.01	a A
		4T	0.80	±0.03	a A	0.81	±0.013	a A	0.80	±0.001	a A
Fv’/Fm’		0.5T	0.69	±0.02	a A	0.68	±0.07	a A	0.68	±0.04	a A
		1T	0.69	±0.04	a A	0.66	±0.06	a A	0.68	±0.003	a A
		2T	0.67	±0.03	a A	0.69	±0.02	a A	0.70	±0.01	a A
		4T	0.70	±0.02	a A	0.64	±0.05	a A	0.63	±0.02	a A
PhiPSII		0.5T	0.56	±0.05	a A	0.51	±0.11	a A	0.58	±0.04	a A
		1T	0.57	±0.05	a A	0.48	±0.08	a A	0.51	±0.01	a A
		2T	0.55	±0.04	a A	0.52	±0.05	a A	0.53	±0.07	a A
		4T	0.64	±0.01	a A	0.48	±0.11	a A	0.45	±0.07	a A
qP		0.5T	0.81	±0.05	a A	0.82	±0.09	a A	0.85	±0.03	a A
		1T	0.82	±0.04	a A	0.72	±0.06	a A	0.75	±0.02	a A
		2T	0.83	±0.03	a A	0.75	±0.05	a A	0.76	±0.11	a A
		4T	0.89	±0.02	a A	0.75	±0.11	a A	0.71	±0.13	a A
qN		0.5T	0.52	±0.11	a A	0.52	±0.14	a A	0.53	±0.09	a A
		1T	0.52	±0.11	a A	0.61	±0.09	a A	0.55	±0.08	a A
		2T	0.55	±0.09	a A	0.57	±0.02	a A	0.53	±0.02	a A
		4T	0.45	±0.02	a A	0.56	±0.13	a A	0.64	±0.04	a A

Net photosynthetic rate (Pn), transpiration rate (Tr), electron transport rate (ETR), maximum quantum yield of PSII primary photochemistry (Fv/Fm), efficiency of excitation energy captured by open PSII reaction centers (F′v/F′m), quantum yield of PSII electron transport (PhiPSII), photochemical quenching (qP), and nonphotochemical quenching (qN) of basil plants at 20 days after transplanting under different ANQ treatments and R:B ratios. 0.5T, 1T, 2T, and 4T represent 4 different ANQ treatments. RB3:7, RB7:3, and RB9:1 represent R:B ratios of 7:3, 3:7, and 9:1, respectively. ^z^ Each value is the mean ± SE of three replicates. Based on Tukey’s new multiple range test at *p* < 0.05. The same lowercase letter means that there is no significant difference among different ANQ treatments, and the same capital letter means that there is no significant difference among different R:B ratios.

## Data Availability

Not applicable.

## Supplementary Materials

The following supporting information can be downloaded at: https://www.mdpi.com/article/10.3390/biology11070991/s1, Figure S1: Spectral distribution of the white LED lamp used in the seedling stage; Table S1: Growth parameters of basil plants grown under ANQ treatments and R:B ratios; Table S2: Analysis of the correlation among shoot dry weight, leaf area, and photosynthesis parameters.
