# Supplementary material for: Growth and Nutrient Utilization in Basil Plant as Affected by Applied Nutrient Quantity in Nutrient Solution and Light Spectrum
Source: Biology (Basel). 2022 Jun 29;11(7):991. doi: 10.3390/biology11070991 (PMC9311585; doi:10.3390/biology11070991)
Supplement: Supplementary file 1 [file biology-11-00991-s001.zip › biology-1764383-SM.pdf]

Growth and nutrient utilization in basil plant as affected by applied nutrient quantity in nutrient solution and light spectrum

Xiaowei Ren [1], Na Lu [2,*], Wenshuo Xu [1], Yunfei Zhuang [1], Satoru Tsukagoshi [2] and Michiko Takagaki [1]

[1]Graduate School of Horticulture, Chiba University, 648, Matsudo 271-8510, Chiba, Japan.
[2]Center for Environment, Health, and Field Sciences, Chiba University, 6-2-1 Kashiwanoha, Kashiwa 277-0882, Chiba, Japan.

Table of Contents

Supplementary Figure S1 Spectral distribution of the white LED lamp used in the seedling stage........2

Supplementary Table S1 Growth parameters of basil plants grown under ANQ treatments and R:B ratios........3

Supplementary Table S2 Analysis of the correlation among shoot dry weight, leaf area and photosynthesis parameters........4

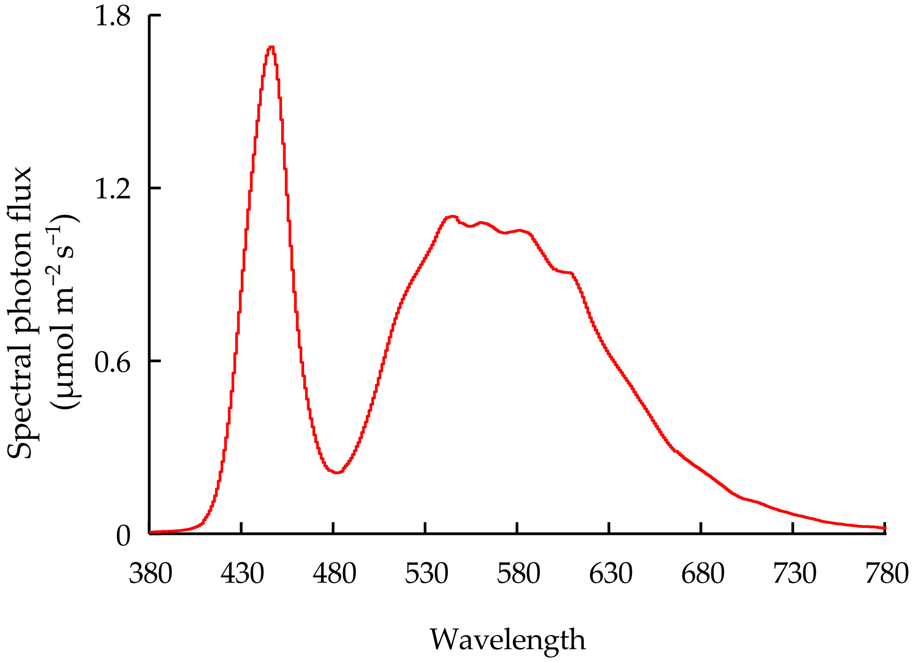

Supplementary Figure S1. Spectral distribution of the white LED lamp used in the seedling stage.

Supplementary Table S1. Growth parameters of basil plants grown under ANQ treatments and R:B ratios.

Growth parameters	Unit	Applied nutrient amount	R:B ratios								
			RB3:7			RB7:3			RB9:1		
Leaf FW	(g/plant)	0.5T	8.22	±1.19	a B	9.83	±0.42	a A	7.98	±1.05	b B
		1T	7.68	±0.95	a C	12.19	±1.14	a A	9.19	±0.53	ab B
		2T	9.68	±1.87	a A	11.80	±1.24	a A	9.72	±1.01	a A
		4T	9.03	±2.65	a A	11.11	±2.39	a A	9.16	±1.03	ab A
Leaf DW	(g/plant)	0.5T	0.62	±0.08	a B	0.79	±0.03	a A	0.66	±0.08	a B
		1T	0.58	±0.08	a C	0.89	±0.08	a A	0.75	±0.07	a B
		2T	0.71	±0.14	a A	0.87	±0.09	a A	0.77	±0.09	a A
		4T	0.75	±0.24	a A	0.87	±0.16	a A	0.77	±0.09	a A
Root FW	(g/plant)	0.5T	6.71	±0.90	a A	6.71	±0.57	b A	6.34	±0.93	a A
		1T	7.65	±0.94	a AB	8.70	±1.33	a A	6.82	±0.53	a B
		2T	8.35	±0.65	a A	8.32	±1.41	ab A	7.58	±0.85	a A
		4T	7.02	±1.59	a A	8.29	±1.35	ab A	7.02	±1.07	a A
Root DW	(g/plant)	0.5T	0.31	±0.03	a B	0.36	±0.03	a A	0.35	±0.03	a B
		1T	0.35	±0.04	a A	0.41	±0.05	a A	0.31	±0.03	a A
		2T	0.38	±0.04	a A	0.42	±0.06	a A	0.36	±0.04	a A
		4T	0.37	±0.08	a A	0.43	±0.06	a A	0.36	±0.04	a A

Leaf fresh weight (FW), leaf dry weight (DW), root FW and root DW of basil plants at 20 days after transplanting under different ANQ treatments and R:B ratios. 0.5T, 1T, 2T and 4T represent 4 different ANQ treatments, respectively. RB3:7, RB7:3, RB9:1 represent R:B ratios of 7:3, 3:7 and 9:1, respectively. The error bars represent SEs (n = 6). Based on Tukey's new multiple range test at $P < 0.05$. The same lowercase letters mean that there is no significant difference among different ANQ treatments, and same capital letters mean that there is no significant difference among different R:B ratios.

Supplementary Table S2. Analysis of the correlation among shoot dry weight, leaf area and photosynthesis parameters.

Parameters		Leaf area	Pn	Tr	Fv/Fm	Fv'/Fm'	PhiPSII	qP	qN	ETR
Shoot dry weight	Correlation coefficient	0.86	0.14	0.03	0.03	0.12	0.11	0.10	0.08	0.12
	P value	0.00	0.41	0.85	0.88	0.47	0.51	0.58	0.66	0.48

Pn, Tr, ETR, Fv/Fm), F′v/F′m, PhiPSII, qP, and qN represent Net photosynthetic rate, transpiration rate, electron transport rate, maximum quantum yield of PSII primary photochemistry, efficiency of excitation energy captured by open PSII reaction centers, quantum yield of PSII electron transport, photochemical quenching, and the nonphotochemical quenching, respectively.
